# Postnatal Hypertension Management and Discharge Documentation: A Retrospective Clinical Audit at a UK University Hospital

**DOI:** 10.7759/cureus.116894

**Published:** 2026-09-25

**Authors:** Ikran Mohamud, Mena Abdalla, Huma Farooq

**Affiliations:** 1 Obstetrics and Gynaecology, Princess Royal University Hospital, London, GBR; 2 Medical Education, Queen's University Belfast, Belfast, GBR; 3 Obstetrics and Gynaecology, King's College Hospital NHS Foundation Trust, London, GBR

**Keywords:** blood pressure monitoring, clinical audit, hypertensive disorders, postnatal care, postpartum hypertension, quality improvement

## Abstract

Background

Postnatal hypertension requires timely blood pressure surveillance, treatment review, safety netting, and communication between maternity and community services. This audit assessed postnatal hypertension care and discharge documentation against local Trust standards at Princess Royal University Hospital.

Methods

A retrospective case-note audit included women with postnatal hypertension who delivered between April and September 2025. For audit eligibility, postnatal hypertension was defined as a pregnancy-related or chronic hypertensive disorder identified through prespecified Epic diagnosis filters and requiring postnatal surveillance. The clinical blood pressure threshold used for the audit was systolic blood pressure of at least 140 mmHg and/or diastolic blood pressure of at least 90 mmHg from birth through six weeks postpartum. Of 150 identified notes, 148 were included after exclusion of one pre-viable birth before 24+0 weeks and one immediate post-delivery transfer to another Trust. Statistical analyses were performed using R version 4.3.3 (R Foundation for Statistical Computing, Vienna, Austria). Exploratory comparisons across pregnancy-induced hypertension, pre-eclampsia, and chronic hypertension/superimposed pre-eclampsia used Kruskal-Wallis tests for continuous variables and Pearson chi-square statistics with Monte Carlo p-values for categorical variables.

Results

Median maternal age was 34.0 years (IQR 30.0-37.0); 76/148 women (51.4%) had pregnancy-induced hypertension, 50/148 (33.8%) had pre-eclampsia, and 22/148 (14.9%) had chronic hypertension or superimposed pre-eclampsia. A valid discharge blood pressure was recorded in 146/148 records (98.6%); 118/146 (80.8%) were below 140/90 mmHg. Blood-pressure parameter plans, two-week general-practitioner review, and clear discharge communication were documented in 27/148 (18.2%), 34/147 (23.1%), and 16/148 (10.8%) records, respectively. Documentation of a blood pressure parameter plan (Pearson chi-square=8.551, p=0.014) and clear discharge communication (Pearson chi-square=8.349, p=0.013) differed across hypertensive disorder groups. Any postnatal antihypertensive was documented in 109/148 records (73.6%); medication documentation differed across groups (Pearson chi-square=12.669, p=0.001). Community re-referral for raised blood pressure occurred in 12/148 records (8.1%); any documented readmission occurred in 14/148 (9.5%).

Conclusions

Discharge blood pressure documentation, safety netting, and breastfeeding drug safety advice were high, but documentation of individualized blood pressure plans, early general practitioner review, and clear discharge communication were low. An electronic discharge template and prospective re-audit are appropriate quality-improvement interventions.

## Introduction

Hypertensive disorders of pregnancy may persist or present after delivery and require reliable postnatal surveillance, treatment review, and an explicit transfer from maternity to community care. The National Institute for Health and Care Excellence (NICE) recommends postnatal blood pressure monitoring and a care plan that identifies the responsible follow-up provider, frequency of blood pressure checks, medication-adjustment thresholds, and indications for primary care referral [1].

Postpartum care is an ongoing process that requires timely contact, coordinated services, and clear responsibility for chronic-condition follow-up [2]. Postnatal hypertension management also provides an opportunity to identify women needing longer-term cardiovascular risk assessment and primary care follow-up [3]. Remote monitoring can improve early postpartum blood pressure ascertainment, although evidence for effects on severe maternal morbidity remains limited [4,5].

This audit evaluated postnatal hypertension management at Princess Royal University Hospital against local Trust standards. The primary objective was to assess documentation of discharge summaries, medication and surveillance plans, safety netting, breastfeeding drug safety advice, and general practitioner follow-up. The secondary objective was to describe documented post-discharge hypertension-related re-referral and readmission.

## Materials and methods

Study design, setting, and operational definition

This retrospective clinical audit (Audit ID 996) was conducted at Princess Royal University Hospital. The audit population comprised women who delivered between April 2025 and September 2025 and met the audit definition of postnatal hypertension. The data were collected through the Electronic Health Record system, which we use in our hospital; it is called the Epic system.

For this audit, the postnatal period was defined as birth through six weeks after delivery. Postnatal hypertension was operationally defined for eligibility as a pregnancy-related or chronic hypertensive disorder recorded through the prespecified Epic diagnosis filters and requiring postnatal surveillance. The clinical blood pressure threshold used for the audit was systolic blood pressure of at least 140 mmHg and/or diastolic blood pressure of at least 90 mmHg during that period. The threshold aligns with NICE criteria for hypertension, and the six-week time window is consistent with the commonly used postpartum window in the literature [6,7]. A discharge blood pressure below 140/90 mmHg required both a systolic value below 140 mmHg and a diastolic value below 90 mmHg.

Eligible notes were identified through the Epic reporting system using diagnosis filters for hypertension complicating pregnancy, gestational hypertension, pre-eclampsia, eclampsia, and chronic hypertension in pregnancy. As this was a retrospective audit, the diagnosis filter approach identified the cohort; serial blood pressure measurements were not re-abstracted to independently re-screen eligibility. Case notes and discharge documentation were reviewed retrospectively. Of 150 identified case notes, 148 were included. One pre-viable birth before 24+0 weeks and one case transferred to another Trust immediately after delivery were excluded.

Audit standards and variables

The audit assessed local Trust standards for postnatal hypertension care. These included documentation of the hypertension diagnosis on the discharge summary, antihypertensive management, a clear postnatal medication and surveillance plan, discharge blood pressure below 140/90 mmHg, safety netting for relevant symptoms, breastfeeding drug safety advice, community blood pressure monitoring, and general practitioner review at two and six weeks postnatally. The local care pathway also specified blood pressure surveillance and timing of discharge according to hypertensive disorder severity.

The analysis included age, ethnicity, hypertensive disorder, gestational age at diagnosis and delivery, delivery type, length of stay, documented postnatal antihypertensive medicines, inpatient blood pressure measurement frequency, discharge blood pressure, discharge documentation measures, community re-referral for raised blood pressure, and documented readmission. The structured data enabled evaluation of whether methyldopa was documented postnatally, but did not allow reliable assessment of the exact timing of methyldopa cessation. Two original manual review outcomes - hypertension diagnosis not documented and no clear postnatal plan - were retained from the audit presentation. Their manual review denominator was 132 records.

Statistical analysis

Analysis was performed in R version 4.3.3 (R Foundation for Statistical Computing, Vienna, Austria). Continuous variables are reported as median (IQR); categorical variables are reported as n (%). Overall values use the 148-case cohort unless a variable had missing data, for which the available denominator is specified in the relevant table legend.

Prespecified exploratory comparisons were made across three hypertensive disorder groups: pregnancy-induced hypertension (n=76), pre-eclampsia (n=50), and chronic hypertension/superimposed pre-eclampsia (n=22). Kruskal-Wallis tests were used for continuous variables. For categorical variables, Pearson chi-square statistics were calculated and p-values derived using 100,000-resample Monte Carlo simulation because some expected cell counts were sparse. Two-sided p<0.05 was considered statistically significant. These analyses were exploratory, unadjusted, and intended to describe documented care patterns; they do not support causal inference. The two manual review outcomes could not be compared across hypertensive disorder groups because only aggregate numerator/denominator data were retained.

This report was prepared with reference to the Strengthening the Reporting of Observational Studies in Epidemiology (STROBE) principles [6].

Ethics and governance

This work was conducted as a registered clinical audit at Princess Royal University Hospital (Audit ID 996).

## Results

Cohort characteristics

The audit included 148 women. Pregnancy-induced hypertension was recorded in 76/148 women (51.4%), pre-eclampsia in 50/148 (33.8%), chronic hypertension in 12/148 (8.1%), and superimposed pre-eclampsia on chronic hypertension in 10/148 (6.8%). Median age was 34.0 years (IQR 30.0-37.0); 104/148 women (70.3%) were White individuals, and cesarean delivery occurred in 97/148 (65.5%).

Across the three comparison groups, gestational age at diagnosis (Kruskal-Wallis H=19.509, p<0.001), gestational age at delivery (H=27.562, p<0.001), and length of stay (H=12.363, p=0.002) differed. Age (H=5.320, p=0.070), ethnicity (Pearson chi-square=11.583, p=0.161), and delivery type (Pearson chi-square=12.731, p=0.120) did not differ significantly. Table 1 presents all cohort characteristics and test results.

**Table 1:** Cohort characteristics and exploratory comparisons by hypertensive disorder group Values are median (IQR) or n (%). Overall denominator is 148; group denominators are pregnancy-induced hypertension, 76; pre-eclampsia, 50; and chronic hypertension/superimposed pre-eclampsia, 22. Continuous variables were compared using Kruskal-Wallis tests. Categorical variables were compared using Pearson chi-square statistics with 100,000-resample Monte Carlo p-values. Missing data were gestational age at diagnosis (17 records), gestational age at delivery (13 records), and length of stay (one record); all other variables in this table had no missing values.

Characteristic	Overall	Pregnancy-induced hypertension	Pre-eclampsia	Chronic hypertension/superimposed pre-eclampsia	Test statistic	P-value
Age, years, median (IQR)	34.0 (30.0-37.0)	33.5 (30.0-36.0)	34.0 (32.0-37.0)	36.0 (33.2-38.0)	Kruskal-Wallis H=5.320	0.070
Gestational age at diagnosis, weeks, median (IQR)	36.4 (31.6-38.6)	37.4 (33.1-39.7)	35.6 (32.0-37.9)	18.4 (15.1-19.6)	Kruskal-Wallis H=19.509	<0.001
Gestational age at delivery, weeks, median (IQR)	38.3 (37.0-39.3)	39.0 (38.0-40.0)	37.6 (36.4-38.7)	37.0 (36.9-38.9)	Kruskal-Wallis H=27.562	<0.001
Length of stay, days, median (IQR)	2.0 (1.0-4.0)	2.0 (1.0-3.0)	3.0 (2.0-7.0)	2.0 (1.0-4.0)	Kruskal-Wallis H=12.363	0.002
Ethnicity, n (%)						
White individuals	104 (70.3)	58 (76.3)	34 (68.0)	12 (54.5)	Pearson chi-square=11.583	0.161
Black individuals	22 (14.9)	9 (11.8)	9 (18.0)	4 (18.2)		
Asian individuals	12 (8.1)	4 (5.3)	6 (12.0)	2 (9.1)		
Mixed individuals	9 (6.1)	4 (5.3)	1 (2.0)	4 (18.2)		
Other/unclear	1 (0.7)	1 (1.3)	0 (0.0)	0 (0.0)		
Delivery type, n (%)						
Emergency cesarean delivery	58 (39.2)	26 (34.2)	23 (46.0)	9 (40.9)	Pearson chi-square=12.731	0.120
Elective cesarean delivery	23 (15.5)	12 (15.8)	8 (16.0)	3 (13.6)		
Cesarean delivery, urgency unclear	16 (10.8)	5 (6.6)	6 (12.0)	5 (22.7)		
Non-instrumental vaginal delivery	34 (23.0)	23 (30.3)	6 (12.0)	5 (22.7)		
Instrumental vaginal delivery	17 (11.5)	10 (13.2)	7 (14.0)	0 (0.0)		

Audit criteria and discharge documentation

A valid discharge blood pressure was available in 146/148 records (98.6%); 118/146 (80.8%) were below 140/90 mmHg. The original manual documentation review found that the hypertension diagnosis was not documented in 56/132 records (42.4%) and that 73/132 records (55.3%) lacked a clear postnatal plan.

Safety netting advice was documented in 146/148 records (98.6%) and breastfeeding drug safety advice in 145/148 (98.0%). A blood pressure parameter plan was documented in 27/148 records (18.2%); general practitioner review at two weeks in 34/147 (23.1%); and clear discharge communication in 16/148 (10.8%). Documentation of a blood pressure parameter plan differed across groups (Pearson chi-square=8.551, p=0.014), as did clear discharge communication (Pearson chi-square=8.349, p=0.013). Other structured discharge criteria did not show statistically significant group differences. Table 2 presents all audit criteria; figures were removed to avoid duplicating tabular data.

**Table 2:** Postnatal hypertension audit criteria and exploratory comparisons by hypertensive disorder group Values are n (%). Overall denominator is 148; group denominators are pregnancy-induced hypertension, 76; pre-eclampsia, 50; and chronic hypertension/superimposed pre-eclampsia, 22. The original manual review outcomes use a denominator of 132 and lack retained subgroup data; therefore, group comparisons, test statistics, and p-values are not applicable for these two rows. For R-derived measures, discharge blood pressure below 140/90 mmHg had two missing values, and two-week general practitioner review had one missing value; all other R-derived measures had no missing values. Pearson chi-square statistics and 100,000-resample Monte Carlo p-values are reported for eligible categorical comparisons.

Audit criterion	Overall	Pregnancy-induced hypertension	Pre-eclampsia	Chronic hypertension/superimposed pre-eclampsia	Test statistic	P-value
Hypertension diagnosis not documented (original manual review)	56 (42.4)	Not available	Not available	Not available	Not applicable	Not applicable
No clear postnatal plan (original manual review)	73 (55.3)	Not available	Not available	Not available	Not applicable	Not applicable
Discharge blood pressure available	146 (98.6)	74 (97.4)	50 (100.0)	22 (100.0)	Pearson chi-square=1.921	0.653
Discharge blood pressure <140/90 mmHg	118 (80.8)	64 (86.5)	37 (74.0)	17 (77.3)	Pearson chi-square=3.212	0.193
Blood pressure parameter plan documented	27 (18.2)	7 (9.2)	14 (28.0)	6 (27.3)	Pearson chi-square=8.551	0.014
Safety-netting advice documented	146 (98.6)	74 (97.4)	50 (100.0)	22 (100.0)	Pearson chi-square=1.921	0.652
Breastfeeding drug safety advice documented	145 (98.0)	73 (96.1)	50 (100.0)	22 (100.0)	Pearson chi-square=2.901	0.281
General-practitioner review at two weeks documented	34 (23.1)	15 (20.0)	11 (22.0)	8 (36.4)	Pearson chi-square=2.616	0.272
Clear discharge communication documented	16 (10.8)	3 (3.9)	8 (16.0)	5 (22.7)	Pearson chi-square=8.349	0.013

Postnatal antihypertensive treatment and inpatient surveillance

A postnatal antihypertensive medication was documented in 109/148 records (73.6%) and no medication in 39/148 (26.4%). Labetalol was the most frequently documented medication (65/148, 43.9%), followed by nifedipine (46/148, 31.1%) and enalapril (10/148, 6.8%); medication categories were not mutually exclusive. No postnatal methyldopa documentation was identified. Four-hourly blood pressure measurement was the most frequently documented inpatient schedule (114/148, 77.0%).

The proportion with any documented postnatal antihypertensive differed across groups (Pearson chi-square=12.669, p=0.001), as did nifedipine documentation (Pearson chi-square=17.168, p<0.001). Labetalol, enalapril, amlodipine, and atenolol documentation did not differ statistically across groups; the global distribution of inpatient measurement frequency also did not differ (Pearson chi-square=10.994, p=0.539). Table 3 provides the full medication and surveillance results. No separate figures are presented because the same data are tabulated.

**Table 3:** Documented postnatal antihypertensive treatment and inpatient blood pressure surveillance, with exploratory comparisons by hypertensive disorder group Values are n (%). Overall denominator is 148; group denominators are pregnancy-induced hypertension, 76; pre-eclampsia, 50; and chronic hypertension/superimposed pre-eclampsia, 22. There were no missing data for variables. Medication categories are not mutually exclusive. Pearson chi-square statistics and 100,000-resample Monte Carlo p-values are reported for binary treatment measures and for the global blood pressure frequency distribution. No test was performed for methyldopa because all observed values were zero.

Treatment or surveillance measure	Overall	Pregnancy-induced hypertension	Pre-eclampsia	Chronic hypertension/superimposed pre-eclampsia	Test statistic	P value
Postnatal antihypertensive documented	109 (73.6)	48 (63.2)	39 (78.0)	22 (100.0)	Pearson chi-square=12.669	0.001
No postnatal antihypertensive documented	39 (26.4)	28 (36.8)	11 (22.0)	0 (0.0)		
Postnatal medicines documented (categories not mutually exclusive)						
Nifedipine	46 (31.1)	12 (15.8)	23 (46.0)	11 (50.0)	Pearson chi-square=17.168	<0.001
Labetalol	65 (43.9)	28 (36.8)	23 (46.0)	14 (63.6)	Pearson chi-square=5.106	0.077
Methyldopa	0 (0.0)	0 (0.0)	0 (0.0)	0 (0.0)	Not applicable	Not applicable
Enalapril	10 (6.8)	3 (3.9)	5 (10.0)	2 (9.1)	Pearson chi-square=1.977	0.402
Amlodipine	4 (2.7)	1 (1.3)	3 (6.0)	0 (0.0)	Pearson chi-square=3.234	0.284
Atenolol	1 (0.7)	0 (0.0)	0 (0.0)	1 (4.5)	Pearson chi-square=5.766	0.150
Documented inpatient blood-pressure measurement frequency						
Every 1 hour	3 (2.0)	1 (1.3)	2 (4.0)	0 (0.0)	Pearson chi-square=10.994	0.539
Every 2 hours	4 (2.7)	2 (2.6)	1 (2.0)	1 (4.5)		
Every 3 hours	1 (0.7)	0 (0.0)	1 (2.0)	0 (0.0)		
Every 4 hours	114 (77.0)	55 (72.4)	41 (82.0)	18 (81.8)		
Every 6 hours	19 (12.8)	12 (15.8)	4 (8.0)	3 (13.6)		
Every 8 hours	5 (3.4)	5 (6.6)	0 (0.0)	0 (0.0)		
Other/unclear	2 (1.4)	1 (1.3)	1 (2.0)	0 (0.0)		

Post-discharge outcomes

Community re-referral for raised blood pressure was documented in 12/148 records (8.1%), with no statistically significant difference across groups (Pearson chi-square=0.507, p=0.775). Any documented readmission occurred in 14/148 records (9.5%) and differed across groups (Pearson chi-square=7.293, p=0.026). These p-values are exploratory and unadjusted; the readmission analysis includes all recorded readmission reasons and does not identify hypertension-specific readmission (Table 4).

**Table 4:** Documented post-discharge outcomes and exploratory comparisons by hypertensive disorder group Values are n (%). The overall denominator is 148; group denominators are pregnancy-induced hypertension, 76; pre-eclampsia, 50; and chronic hypertension/superimposed pre-eclampsia, 22. There were no missing data. Pearson chi-square statistics and 100,000-resample Monte Carlo p-values are shown.

Post-discharge outcome	Overall	Pregnancy-induced hypertension	Pre-eclampsia	Chronic hypertension/superimposed pre-eclampsia	Test statistic	P-value
Community re-referral for raised blood pressure	12 (8.1)	5 (6.6)	5 (10.0)	2 (9.1)	Pearson chi-square=0.507	0.775
Any documented readmission	14 (9.5)	5 (6.6)	9 (18.0)	0 (0.0)	Pearson chi-square=7.293	0.026

## Discussion

Principal findings

This retrospective clinical audit of 148 women with postnatal hypertension found high documentation of discharge blood pressure, safety-netting advice, and breastfeeding drug safety advice. However, several transition-of-care measures were inconsistently documented. In the original manual review, 42.4% of assessed records did not document the hypertension diagnosis and 55.3% did not contain a clear postnatal plan. The structured R reanalysis identified low documentation of blood pressure parameter plans (18.2%), two-week general practitioner review (23.1%), and clear discharge communication (10.8%).

The audit found that 80.8% of records with a valid discharge blood pressure were below 140/90 mmHg. Postnatal antihypertensive medication was documented in nearly three-quarters of the cohort, with labetalol and nifedipine most frequently recorded; no postnatal methyldopa was documented. Four-hourly monitoring was the predominant inpatient blood pressure schedule. Community re-referral for raised blood pressure occurred in 8.1% of the cohort, and any recorded readmission in 9.5%.

Exploratory comparisons showed differences in the timing of hypertension diagnosis, delivery gestation, and length of stay across hypertensive disorder groups. Documentation of blood pressure parameter plans and clear discharge communication also differed across groups. Medication documentation and nifedipine use varied by group, while the global distribution of inpatient blood pressure frequency did not. These exploratory unadjusted comparisons should not be interpreted as evidence of causal differences in care quality or outcomes.

Interpretation and implications for practice

NICE recommends a postnatal care plan that identifies the follow-up provider, blood pressure monitoring frequency, medication management thresholds, and criteria for primary care referral [1]. The gaps identified in this audit directly affect the continuity elements expected during transfer from hospital to community care. Optimizing postpartum care requires timely follow-up, care coordination, and explicit responsibility for ongoing management [2].

The local action plan should focus on an electronic discharge smartphrase that prompts clinicians to document the hypertension diagnosis, current medication and any titration or cessation plan, blood pressure monitoring parameters, safety-netting advice, breastfeeding drug safety advice, and planned general practitioner review. For women with chronic hypertension or severe pre-eclampsia, the local pathway should facilitate referral to the appropriate postnatal blood pressure clinic at six weeks. Targeted education for junior doctors and a repeat audit after implementation can determine whether the intervention improves documentation reliability.

Postpartum home blood pressure monitoring and text-based monitoring may improve early blood pressure ascertainment [4,5]. Their implementation should be considered alongside local staffing, access, patient preference, and equitable engagement, rather than assumed to replace the discharge documentation and communication processes assessed in this audit.

Strengths and limitations

This audit used a defined hospital population, prespecified audit period, electronic case identification, and final cohort of 148 records. It was independently reanalysed in R after author-confirmed exclusions. The revised analysis reports all prespecified variables, test statistics, p-values, available denominators, and the original manual review denominator for its two aggregate documentation outcomes.

The audit was retrospective and single-centre, so findings may not generalize to other services. Documentation quality may differ from the clinical care delivered, and post-discharge events recorded outside the available record may not have been captured. Two original manual review outcomes had an aggregate denominator of 132 and could not be compared by hypertensive disorder subgroup. Recorded readmission included all causes, so hypertension-specific readmission could not be estimated. The structured data did not permit reliable assessment of the timing of methyldopa cessation. Multiple exploratory unadjusted group comparisons were performed; some groups and outcome cells were small, and the analysis does not support causal inference.

## Conclusions

At Princess Royal University Hospital, this postnatal hypertension audit found high documentation of discharge blood pressure, safety netting, and breastfeeding drug-safety advice. The principal quality improvement opportunity is a reliable discharge process that documents the hypertension diagnosis, individualized medication and blood pressure surveillance plan, early general practitioner review, and clear communication with community teams.

An electronic discharge smartphrase, focused staff education, and prospective re-audit are appropriate next steps to improve consistency and support safe continuity of postnatal hypertension care.
